# Burden of colon and rectum cancer attributable to a diet high in red meat in the United States, 1990–2021

**DOI:** 10.3389/fnut.2026.1683427

**Published:** 2026-03-24

**Authors:** Liang Huang, Minghua Li

**Affiliations:** Department of Gastroenterology, The Fourth People's Hospital of Longgang District, Shenzhen, China

**Keywords:** age-standardized rates, colon and rectum cancer, global burden of disease, red meat, United States

## Abstract

**Background:**

Colon and rectum cancer (CRC) remains a major cause of cancer mortality in the United States. Within the Global Burden of Disease (GBD) framework, high intake of red meat has been modeled as a dietary risk factor for CRC; however, population-level estimates of the associated CRC burden across time, age groups, and United States remain incompletely characterized.

**Methods:**

Using data from GBD 2021, we quantified CRC deaths and disability-adjusted life years (DALYs) attributable to high intake of red meat, as defined by the GBD comparative risk assessment framework, in the United States from 1990 to 2021. Trends in age-standardized mortality rates (ASMR) and DALY rates (ASDR) were evaluated using estimated annual percentage change (EAPC). State-level variation and age-specific patterns were also examined based on modeled population-level estimates.

**Results:**

In 2021, an estimated 12,053 CRC deaths were attributable to high red meat intake in the United States according to GBD modeling, with declining ASMR and ASDR (EAPCs: −1.69 and −1.38, respectively). Males exhibited higher ASMR (2.45 vs. 1.72) and ASDR (61.72 vs. 41.82) than females. California, Texas, and Florida accounted for the highest absolute numbers of deaths, whereas Mississippi, Louisiana, and West Virginia showed the highest age-standardized rates. Age-stratified analyses indicated increasing mortality rates among adults aged 25–49 years (EAPC for ASMR: 0.17), contrasting with declining trends in older age groups. Decomposition analysis suggested that population growth was the dominant contributor to increases in absolute mortality.

**Conclusion:**

Although age-standardized CRC mortality and DALY rates attributable to high red meat intake have declined at the population level, the rising burden among younger adults and persistent geographic disparities across states remain concerning. As these findings are derived from ecological, model-based GBD estimates and do not reflect individual-level associations or causal effects, they should be interpreted with caution. Further research using individual-level data and more comprehensive dietary risk assessments, including processed meat and other dietary factors, is warranted.

## 1 Introduction

Cancer is a leading cause of death globally, and its burden continues to increase. In the United States (U.S.), cancer is the second leading cause of death. According to the latest estimates from the American Cancer Society, approximately 2 million new cancer cases and 610,000 cancer-related deaths are projected in 2024, imposing a substantial economic burden expected to reach $246 billion by 2030 (1, 2). Among specific cancer types, colon and rectum cancer (CRC) ranks as the second leading cause of cancer-related mortality, responsible for 53,010 deaths, making it a major public health challenge (3). While overall CRC incidence in the U.S. has declined over recent decades, rates among adults under 55 years have increased steadily, a trend that remains incompletely explained. A recent study examining early-onset CRC incidence among adults aged 20–44 reported a persistent upward trajectory, highlighting the need to better characterize modifiable lifestyle and dietary contributors (4).

CRC etiology is multifactorial, with dietary patterns playing a central role alongside behavioral, metabolic, and environmental factors. Red meat represents an important source of protein and micronutrients in many diets (5); however, excessive intake has been consistently linked to adverse health outcomes. Despite long-standing dietary recommendations advocating moderation of animal-based foods, global trade and consumption of red and processed meats have increased substantially, contributing to the rising burden of diet-related non-communicable diseases. According to Global Burden of Disease (GBD) 2019 estimates, approximately 32% of CRC deaths were attributable to dietary risks (6). A growing body of epidemiological evidence supports an association between high red and processed meat intake and elevated CRC risk (7, 8), with meta-analyses indicating that each additional 100 g/day of red meat consumption is associated with increased cancer risk (9).

The U.S. has the highest per capita meat consumption globally—more than three times the global average—with red meat accounting for approximately 58% of total meat intake (10, 11). Importantly, meat consumption patterns in the U.S. vary substantially across states, reflecting differences in food environments, cultural norms, socioeconomic conditions, and access to healthy food options. States with higher levels of food insecurity, limited availability of fresh foods, and greater reliance on energy-dense animal-based diets may therefore experience a disproportionate diet-attributable CRC burden. The Dietary Guidelines for Americans, 2020–2025 explicitly recommend reducing red meat consumption to promote long-term health (12), yet adherence to these recommendations remains heterogeneous across regions.

To our knowledge, no previous study has leveraged the most recent GBD 2021 data to provide a longitudinal, age- and state-specific assessment of CRC burden attributable specifically to high red meat intake in the U.S. To address this gap, we used the most recent GBD 2021 data to quantify national and subnational trends in colorectal cancer deaths and disability-adjusted life years attributable to a diet high in red meat in the U.S. from 1990 to 2021. By integrating age-, sex-, and state-level analyses, this study aims not only to characterize geographic and demographic disparities in diet-attributable colorectal cancer burden, but also to provide population-level evidence that may inform the prioritization of dietary risk reduction efforts, colorectal cancer screening strategies, and state-level public health planning.

## 2 Methods

### 2.1 Data sources

The GBD study is a large-scale international collaborative effort involving over 12,000 researchers from more than 160 countries and regions. GBD 2021, the most recent iteration, provides specific estimates for 88 risk factors from 1990 to 2021 at the global, regional, and national levels (204 countries and territories). It employs a comprehensive methodology to calculate risk-specific exposures, relative risks, theoretical minimum risk exposure levels (TMRELs), and population attributable fractions to assess disease burden attributable to various risk factors. Detailed computational methods have been described extensively in prior publications (13).

Consistent with previous GBD iterations, GBD 2021 evaluated a range of behavioral, environmental, occupational, and metabolic risks. Risk-outcome pairs were validated based on rules of evidence, categorized into four hierarchical levels. Diet high in red meat falls under Tier 3 of Dietary risks, which itself is a Tier 2 subcategory under Tier 1 Behavioral risks. Dietary data in GBD 2021 were sourced from multiple inputs, including nationally representative nutrition surveys using 24 h dietary recall, food frequency questionnaires, household budget surveys, Euromonitor national sales data, and Food and Agriculture Organization food supply datasets. Data from 24 h recall surveys were prioritized as the primary source. Mean intake levels for each dietary risk factor were estimated using a spatiotemporal Gaussian process regression (ST-GPR) framework, with standard deviations derived via regression equations. For diet high in red meat, a J-shaped risk curve was applied to determine theoretical minimum risk exposure levels (TMREL), with modeling details described in published literature (13). The 95% uncertainty interval (UI) is represented by the 2.5th and 97.5th percentiles during the 500 iterations of calculating the final estimate.

### 2.2 Define

In GBD 2021, CRC was defined as malignant neoplasms of the colon and rectum (ICD-10 codes C18–C21). “Diet high in red meat” refers to the average daily consumption (in grams/day) of red meat, specifically including mammalian muscle meats such as beef, pork, lamb, and goat, while explicitly excluding all processed meats, poultry, fish, and eggs. According to the GBD 2021 comparative risk assessment framework, the theoretical minimum risk exposure level (TMREL) for red meat intake was defined as 0–200 g/day (13). This TMREL reflects the exposure range associated with the lowest modeled risk across available epidemiological evidence within the GBD framework, rather than a dietary recommendation. Although this upper bound may appear higher than current public health guidelines, it reflects the modeling assumptions used to estimate attributable burden within the GBD framework; therefore, exposures exceeding 200 g/day were considered associated with increased risk in this study.

Disease burden was quantified using disability-adjusted life years (DALYs), where one DALY represents the loss of 1 year of full health. Total DALYs for a condition combine years of life lost due to premature mortality and years lived with disability (14). The socio-demographic index (SDI), a composite measure of socioeconomic development, categorizes regions into five levels: high, high-middle, middle, low-middle, and low. The U.S. is classified as a high-SDI region.

### 2.3 Study population

Since GBD 2021 modeling begins at age 25, our analysis included individuals aged 25+ years, stratified into 14 age groups (25–29, 30–34,..., 95+). Further aggregation was applied for subgroup analyses (25–49, 50–69, and 70+ years).

### 2.4 Statistical analysis

We assessed the burden of CRC attributable to diet high in red meat in the U.S. at both national and state levels, with additional stratification by age and sex. To account for population differences, age-standardized rates (ASRs) were calculated per 100,000 population using the formula: where A is the total number of age groups, α_i_ is the age-specific rate for the ith group, and ω_i_ represents the number of individuals in the corresponding age group of the standard population (15). To evaluate changes in death counts between 1990 and 2021, relative percentage change was computed as: $Percentage\;change\;=\;\frac{Value_{2021}-Value_{1990}}{Value_{1990}}\;\times \;100\%$. Trends in ASRs from 1990 to 2021 were analyzed using estimated annual percentage change (EAPC), derived from the linear regression model: ln (ASR) = α + β × (calendar year) +ε, EAPC = 100 × [exp (β)−1], where α is the intercept term,ε is the error term. The 95% confidence interval (CI) for EAPC was generated from the standard error of β. An ASR was considered increasing if both the EAPC and its 95% CI were >0, and decreasing if both were < 0. Decomposition analysis was performed to quantify contributions of population growth, aging, and age-specific incidence to changes in deaths attributable to diet high in red meat in the U.S. All analyses and visualizations were conducted in R (version 4.3.2).

## 3 Results

### 3.1 Overall burden

In 2021, an estimated 12,052.6 CRC deaths in the U.S. were attributable to high red meat intake according to GBD 2021 estimates, with ASMR of 2.06 per 100,000 and ASDR of 51.26 per 100,000 (Table 1). From 1990 to 2021, ASMR and ASDR both showed significant declining trends, with EAPCs of −1.69 and −1.38, respectively.

**Table 1:** All-age and age-standardized deaths and DALYs of colon and rectum cancer attribute to diet high in red meat in United States.

**Sex**	**Age**	**Deaths**				**DALYs**	
**Number in 2021 (95% UI)**	**Percent change (%)**	**ASR (95% UI)**	**EAPC (95% CI)**	**ASR (95% UI)**	**EAPC (95% CI)**
Both	All ages	12,052.6 (−5.8 to 24,067.1)	12.8	2.06 (0 to 4.1)	−1.69 (−1.77 to −1.62)	51.26 (−0.03 to 101.79)	−1.38 (−1.45 to −1.32)
	25–49	674.3 (−0.5 to 1,333.4)	23.3	0.59 (0 to 1.17)	0.17 (0.09 to 0.25)	29.67 (−0.02 to 58.89)	0.22 (0.14 to 0.3)
	50–69	4,240.4 (−2.4 to 8,440.2)	21.4	4.87 (0 to 9.65)	−1.58 (−1.68 to −1.48)	157.6 (−0.1 to 312.1)	−1.41 (−1.5 to −1.32)
	70+	7,138 (−2.7 to 14,328)	6.4	18.44 (−0.01 to 37)	−2.03 (−2.14 to −1.92)	275.94 (−0.12 to 549.42)	−2.09 (−2.2 to −1.98)
Female	All ages	5,686.8 (−2.6 to 11,405.6)	4.8	1.72 (0 to 3.43)	−1.71 (−1.79 to −1.63)	41.82 (−0.02 to 83.32)	−1.44 (−1.51 to −1.38)
	25–49	288.6 (−0.2 to 569.8)	17.9	0.5 (0 to 0.99)	0.07 (−0.01 to 0.16)	25.25 (−0.02 to 50.05)	0.13 (0.04 to 0.22)
	50–69	1,683.7 (−1.1 to 3,333)	12.3	3.74 (0 to 7.44)	−1.74 (−1.85 to −1.62)	121.23 (−0.07 to 241.21)	−1.57 (−1.68 to −1.46)
	70+	3,714.5 (−1.2 to 7,547.9)	0.9	16.25 (−0.01 to 32.96)	−1.95 (−2.07 to −1.83)	239.09 (−0.08 to 484.21)	−2.02 (−2.14 to −1.9)
Male	All ages	6,365.8 (−3.2 to 12,668.9)	19.6	2.45 (0 to 4.88)	−1.8 (−1.87 to −1.73)	61.72 (−0.03 to 122.17)	−1.43 (−1.5 to −1.36)
	25–49	385.7 (−0.3 to 769.1)	27.7	0.68 (0 to 1.35)	0.24 (0.15 to 0.33)	34.17 (−0.02 to 67.57)	0.29 (0.2 to 0.38)
	50–69	2,556.7 (−1.6 to 5,100.6)	28.3	6.09 (0 to 12.07)	−1.51 (−1.61 to −1.41)	196.57 (−0.13 to 390.04)	−1.34 (−1.43 to −1.25)
	70+	3,423.5 (−1.5 to 6,822.2)	13.1	21.31 (−0.01 to 42.61)	−2.28 (−2.38 to −2.17)	322.55 (−0.16 to 640.89)	−2.31 (−2.42 to −2.21)

ASR, age-standardized rate; EAPC, estimated annual percentage change; DALYs, disability-adjusted life years; UI, uncertainty interval; CI, confidence interval.

**Figure 1:**
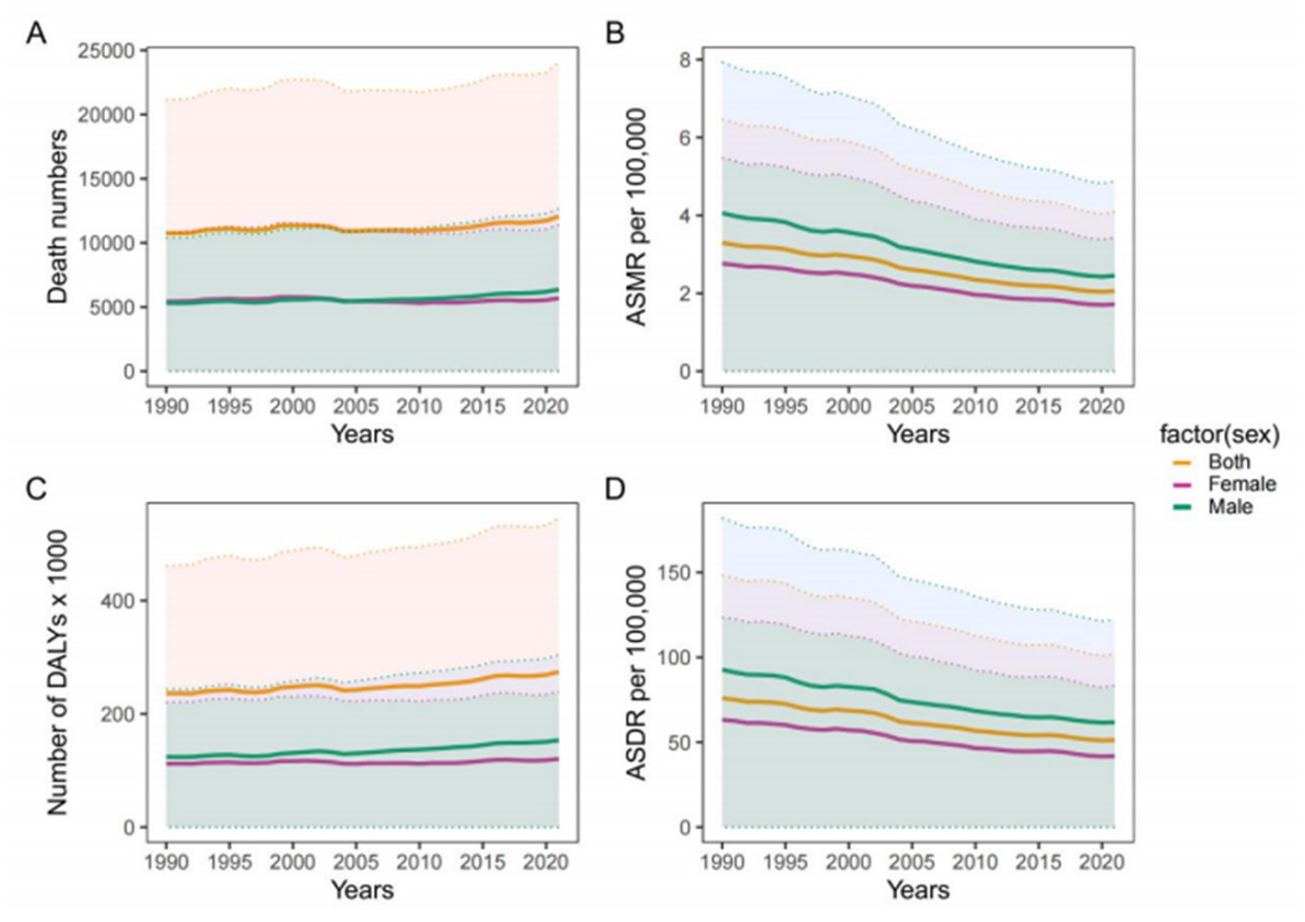
Time trends in CRC burden attributable to diet high in red meat in the United States from 1990 to 2021, shown as number of deaths **(A)**, ASMR **(B)**, number of DALYS **(C)**, and ASDR **(D)**. Although the absolute number of deaths and DALYs increased over time, both ASMR and ASDR declined, illustrating a divergence between population-level burden and per-capita risk. This pattern highlights the growing influence of demographic factors, such as population growth and aging, in shaping the total CRC burden despite improvements in age-standardized outcomes. CRC, colon and rectum cancer; ASMR, age-standardized mortality rate; ASDR, age-standardized DALYs rate; DALYs, disability-adjusted life years.

Over the same period, however, the absolute numbers of CRC deaths and DALYs increased slightly for both sexes combined, as well as for males and females separately (Figure 1). Throughout the study period, males consistently exhibited higher ASMR and ASDR than females. In 2021, male ASMR and ASDR were 2.45 and 61.72, respectively, compared with 1.72 and 41.82 among females (Table 1). Declining trends in age-standardized rates were observed in both sexes.

### 3.2 State-level burden

**Figure 2:**
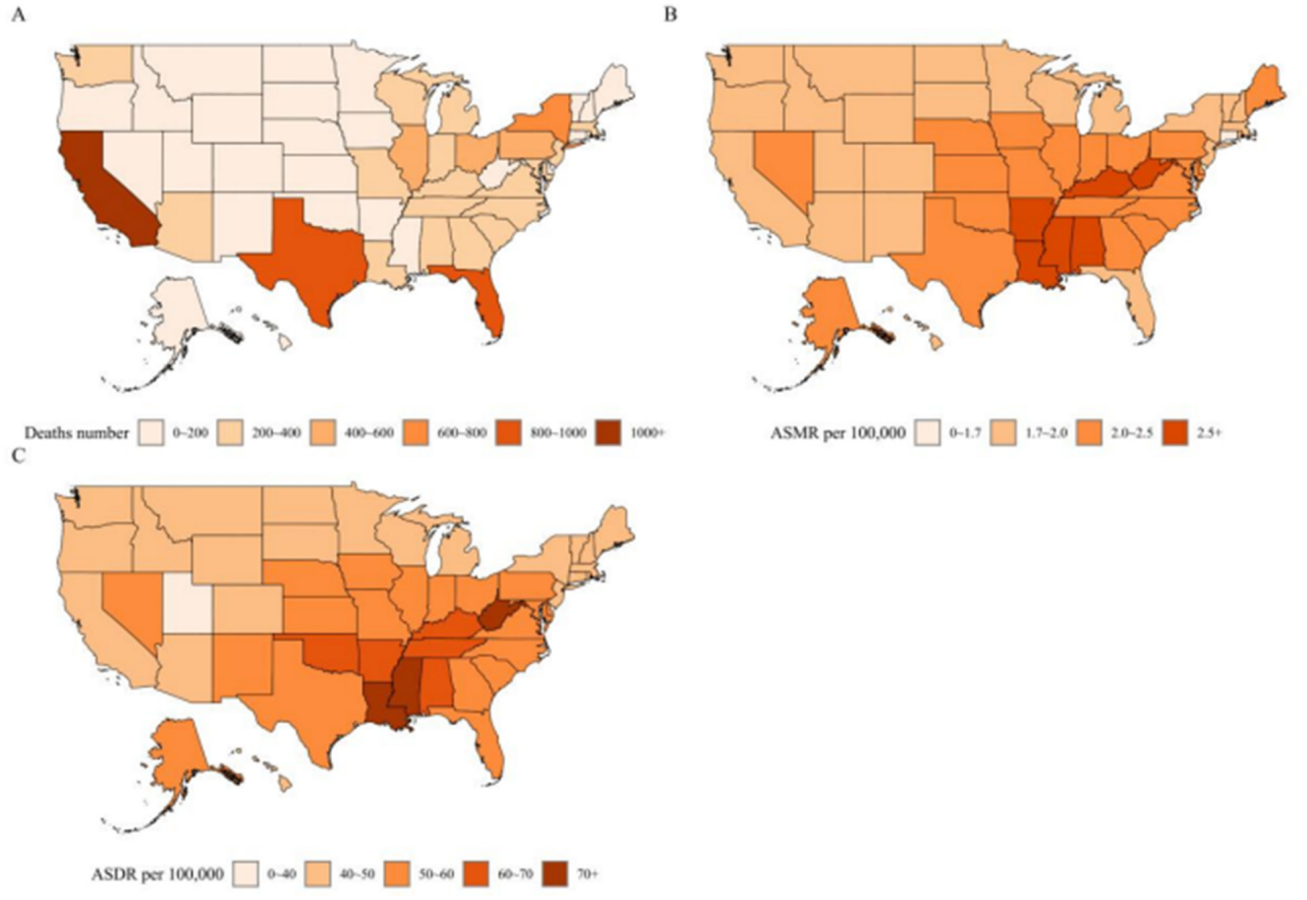
State-level variation in the burden of colon and rectum cancer attributable to diet high in red meat in the United States in 2021. **(A)** Absolute number of deaths attributable to diet high in red meat. **(B)** ASMR per 100,000 population. **(C)** ASDR per 100,000 population. Highly populous states such as California exhibited the largest absolute number of deaths, whereas states in the Deep South, including Mississippi, showed the highest age-standardized mortality and DALY rates. This contrast underscores the distinction between population-level risk and absolute disease burden, with important implications for state-specific public health prioritization and resource allocation. ASMR, age-standardized mortality rate; ASDR, age-standardized DALYs rate; DALYs, disability-adjusted life years.

Substantial variation in CRC burden attributable to high red meat intake was observed across U.S. in 2021 (Figure 2; Supplementary Table 1). California (1,172.2 deaths), Texas (942.3 deaths), and Florida (868.3 deaths) recorded the highest absolute numbers of CRC deaths, whereas Wyoming (20.5 deaths) had the lowest.

In contrast, the highest age-standardized mortality rates were observed in Mississippi (2.96 per 100,000), Louisiana (2.79 per 100,000), and West Virginia (2.72 per 100,000), while Connecticut (1.70 per 100,000) had the lowest ASMR. A similar pattern was observed for ASDR, with the highest rates in Mississippi (77.2 per 100,000), Louisiana (71.0 per 100,000), and West Virginia (70.4 per 100,000), and the lowest in Utah (40.0 per 100,000).

### 3.3 Age and sex patterns

**Figure 3:**
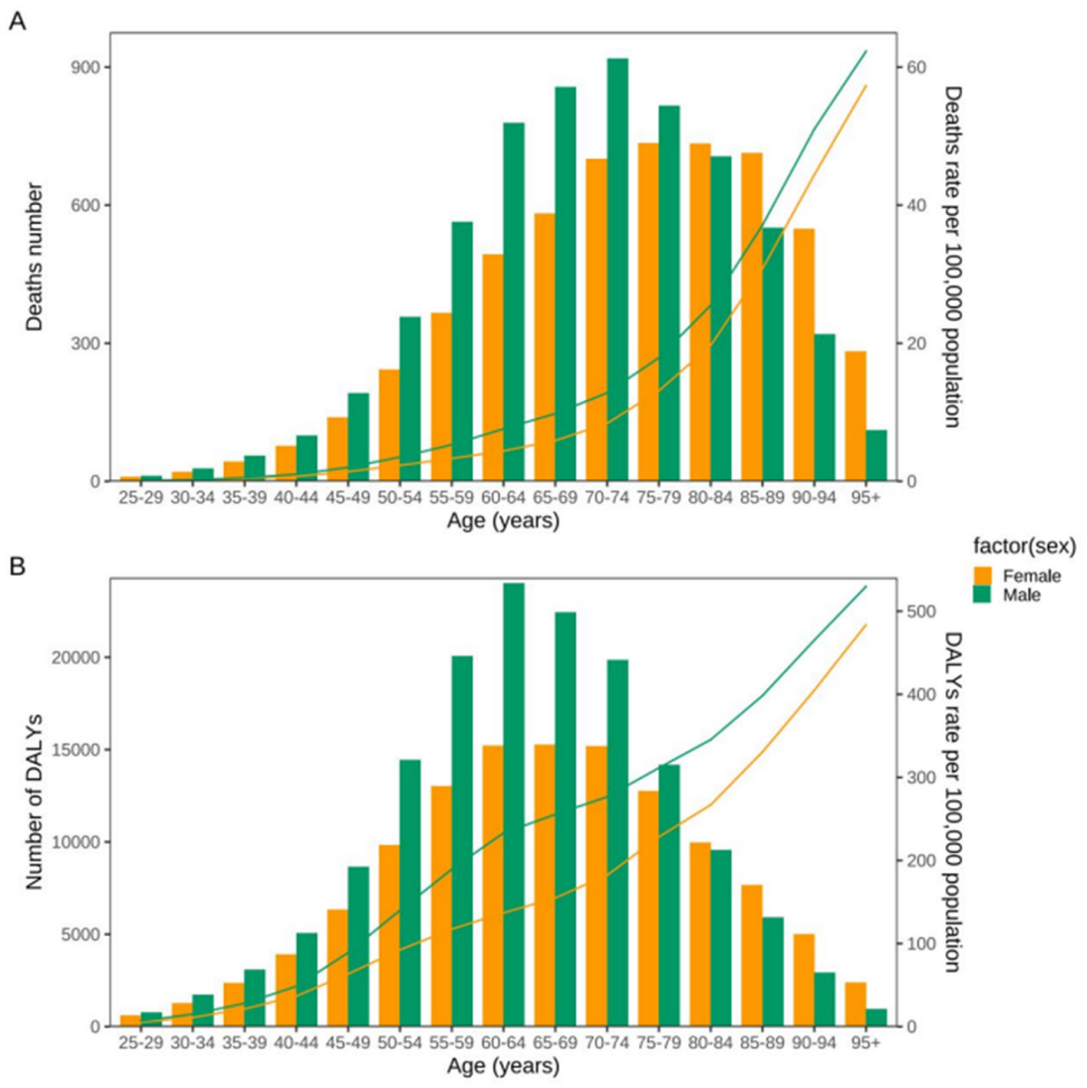
Age-specific distribution of deaths **(A)** and DALYs **(B)** from colon and rectum cancer attributable to diet high in red meat in the United States in 2021. The burden increased markedly with age, peaking in older adults, but substantial heterogeneity was observed across age groups. This age-gradient pattern highlights the concentration of colon and rectum cancer burden in older populations while providing essential context for interpreting divergent temporal trends observed in younger age groups. DALYs, disability-adjusted life years.

In 2021, CRC deaths attributable to high red meat intake increased with age in both sexes, beginning in the 25–29 age group (Figure 3). Among males, deaths peaked in the 70–74 year age group, whereas among females, the peak occurred in the 75–79 year age group. Male deaths exceeded female deaths in most age groups below 75 years, while female deaths were higher in the oldest age groups.

**Figure 4:**
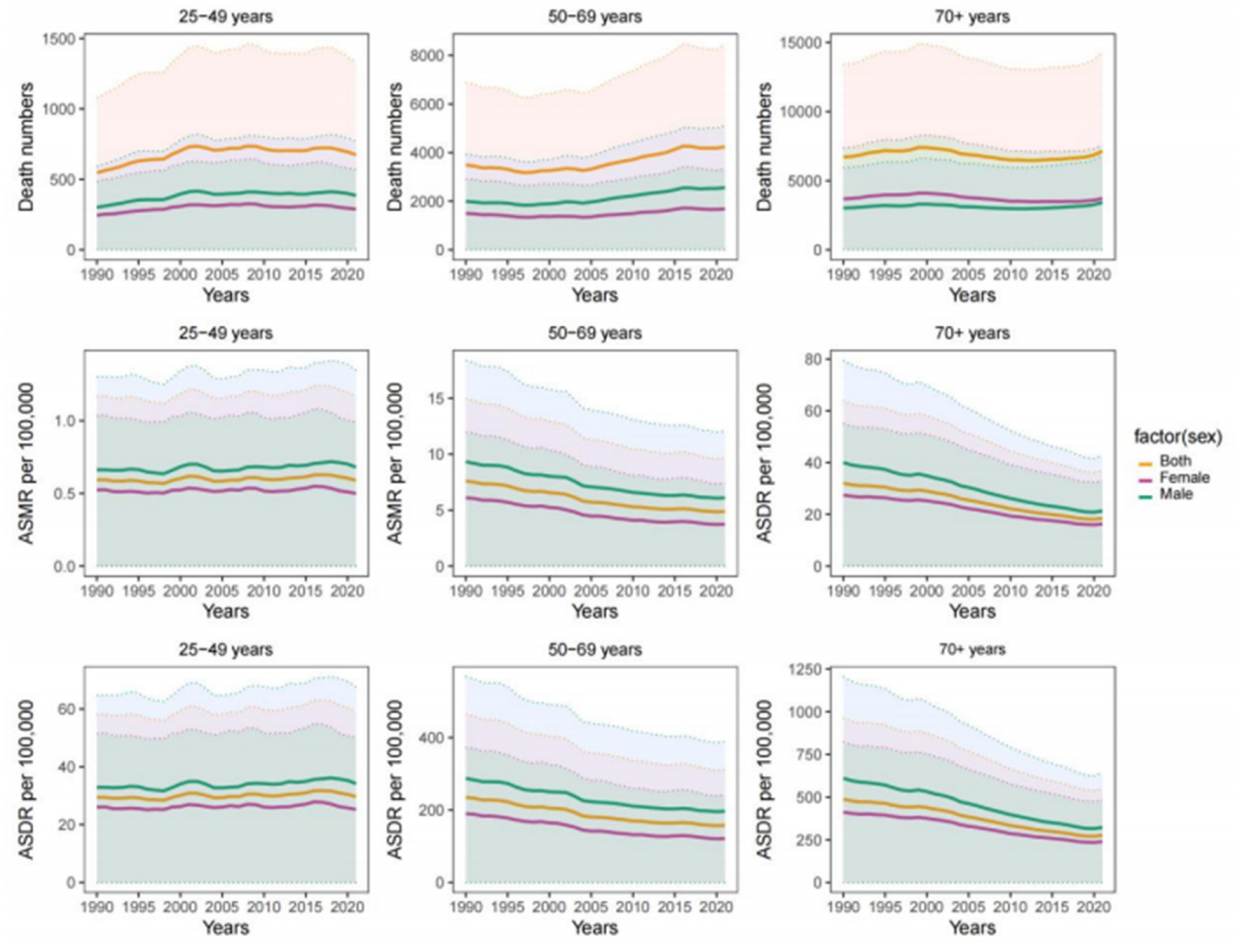
Trends in the number of deaths, ASMR, and ASDR for CRC attributable to diet high in red meat across age groups in the United States from 1990 to 2021. While older age groups accounted for most deaths, age-standardized rates increased among adults aged 25–49, contrasting with declining trends in older populations. This divergence highlights the emerging public health challenge of early-onset CRC that is not apparent from overall trends alone. CRC, colon and rectum cancer; ASMR, age-standardized mortality rate; ASDR, age-standardized DALYs rate; DALYs, disability-adjusted life years.

Age-specific temporal trends differed markedly across age groups (Figure 4; Table 1). Between 1990 and 2021, adults aged 25–49 years experienced increasing age-standardized rates, with EAPCs of 0.17 for ASMR and 0.22 for ASDR for both sexes combined. The increase was more pronounced among males aged 25–49 years (ASMR EAPC 0.24; ASDR EAPC 0.29) than among females of the same age group.

In contrast, both ASMR and ASDR declined in the 50–69 year and ≥70 year age groups, with the largest decreases observed among males aged 70 years and older (ASMR EAPC −2.31). Despite declining age-standardized rates, older age groups continued to account for the majority of absolute CRC deaths and DALYs.

### 3.4 Decomposition analysis

**Figure 5:**
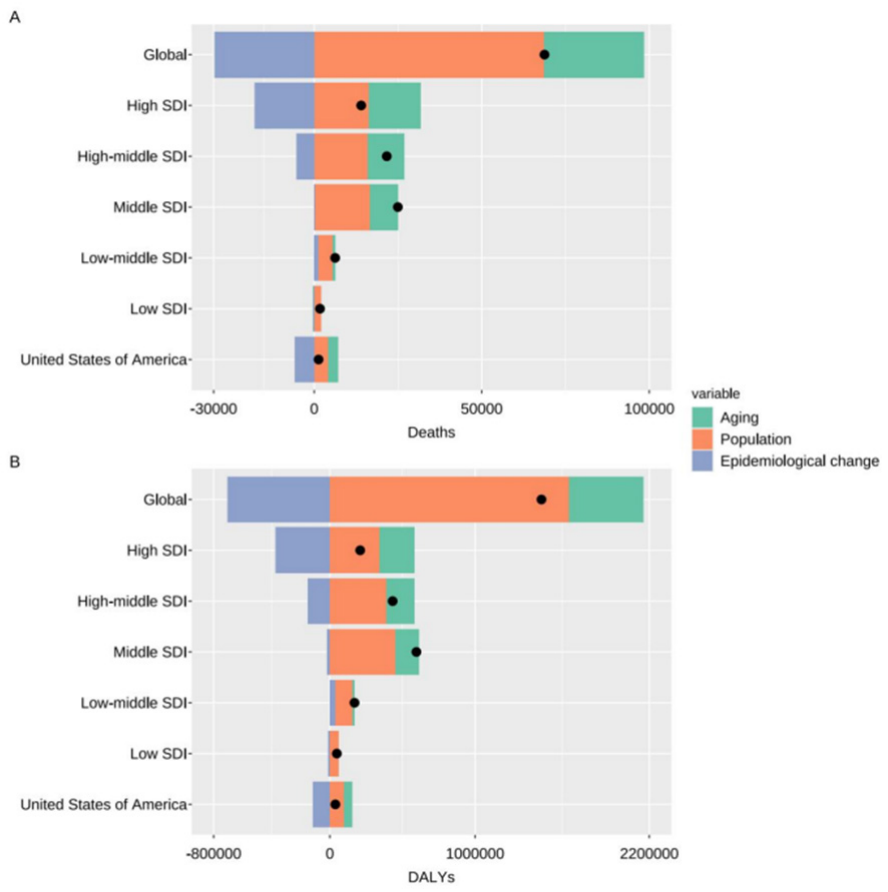
Decomposition of changes in deaths **(A)** and DALYS **(B)** from CRC attributable to diet high in red meat in the United States from 1990 to 2021. Population growth and aging contributed substantially to increases in absolute burden, whereas epidemiological changes exerted a negative effect, partially offsetting these demographic pressures. This decomposition reveals a public health paradox in which improvements in per-capita risk are outweighed by demographic momentum, resulting in a continued rise in total CRC burden. CRC, colon and rectum cancer; DALYS, disability-adjusted life years.

Decomposition analysis of CRC deaths attributable to high in red meat from 1990 to 2021 showed that population growth was the largest contributor to the increase in absolute deaths in the U.S., accounting for 322.68% of the net change for both sexes combined (Figure 5; Supplementary Table 2). Population aging also contributed positively, whereas epidemiological change contributed negatively (−448.51%), partially offsetting the effects of demographic factors.

Comparable decomposition patterns were observed for males and females and for DALYs. Overall, the U.S. pattern closely resembled that of high–sociodemographic index regions, with demographic factors outweighing reductions associated with epidemiological change.

## 4 Discussion

This study provides a comprehensive assessment of the long-term burden of colorectal cancer attributable to high red meat intake in the U.S. and reveals several findings of direct relevance to public health planning. Although age-standardized mortality and DALY rates declined over the past three decades, the absolute burden continued to increase, with pronounced disparities by age, sex, and state. These patterns suggest that population-level improvements in per-capita risk have not translated into proportional reductions in total disease burden and highlight the importance of targeted, rather than uniform, prevention strategies. In particular, the observed heterogeneity across states and age groups underscores the potential value of using subnational burden estimates to guide more tailored dietary and screening interventions.

We found that deaths from CRC attributable to diet high in red meat in the U.S. increased by 12.8% compared to 1990, which can be attributed to the 34% increase in the elderly population since 2012 (16), expanding the high-risk population base. The declines in ASMR and ASDR may be related to reduced risk attributable to diet high in red meat, as the 2015–2020 Dietary Guidelines for Americans explicitly recommended limiting red and processed meat intake. Additionally, colonoscopy screening significantly reduces incidence and mortality rates, especially after the expansion of Medicare coverage in 2001, which increased colonoscopy rates among adults aged 50+ from 20% in 2000 to 61% in 2018. Furthermore, advances in surgical techniques, targeted therapies, and immunotherapy have led to unprecedented progress in cancer treatment over the past decade. For example, by January 2022, cancer survivors accounted for 5% of the U.S. population, compared to just 1.4% fifty years ago (17). However, the observed declines in ASMR and ASDR should be interpreted with caution. Within the GBD framework, these trends represent modeled changes in population-attributable burden rather than direct evidence of reduced individual-level risk. While improvements in CRC screening, treatment, and broader public health efforts—including dietary guidance—may plausibly contribute to declining per-capita risk, part of the observed decline may also reflect modeling assumptions and uncertainty inherent in estimating attributable fractions from heterogeneous exposure distributions. Therefore, these findings should not be interpreted as causal effects but rather as population-level estimates under counterfactual scenarios defined by the GBD methodology.

California had the highest number of deaths from CRC attributable to diet high in red meat, consistent with the latest data published in Cancer Statistics, 2025, which also identifies California as having the highest estimated number of new cases and deaths from CRC in 2025. Mississippi had the highest ASMR and ASDR. These geographic differences reflect not only variations in screening practices and healthcare access, but also broader social determinants of health (18). States like Mississippi and Louisiana–identified as having the highest CRC burden attributable to red meat consumption–also rank among the lowest in socioeconomic indicators such as income, education, and food security. These states have a high prevalence of food deserts, limited access to preventive care, and higher rates of comorbid conditions such as obesity and diabetes. Collectively, these structural disadvantages may exacerbate the impact of dietary risks and limit the effectiveness of risk-reducing behaviors and interventions. Colonoscopy remains the gold standard for CRC screening (19). Studies have found that colonoscopy use among U.S. adults aged 50–75 increased from 2010 to 2018, but rates remained low among unmarried and uninsured adults aged 50–64 (20). The increase in usage may be attributed to expanded insurance coverage and implementation of the Affordable Care Act (21). A 2020 study found California had the lowest screening rate (53%). Although California has implemented the California Colon Cancer Control Program strategies, a recent survey found that without continued funding support, the sustainability of CRC screening cannot be guaranteed (22). Mississippi has the highest CRC mortality rate in the U.S., and food insecurity may be one of the contributing risk factors for CRC (23). A study on factors associated with CRC screening among Mississippi adults found that having insurance coverage or access to healthcare services increased the likelihood of compliance with CRC screening. In addition to differences in healthcare access and screening uptake, state-level variation in CRC burden attributable to high red meat intake may also reflect substantial heterogeneity in dietary behaviors and food environments. States with higher food insecurity, limited access to affordable healthy foods, and cultural norms favoring meat-centered diets may experience greater exposure to red meat-related dietary risks, thereby amplifying the observed geographic disparities. These findings emphasize the need for tailored interventions to strengthen CRC screening across states.

CRC mortality increases with age, and while overall mortality rates are declining, this trend is largely driven by reductions among adults aged 50 years and older. Notably, our analysis identified a significant and concerning increase in age-standardized mortality rates among adults aged 25–49 years, consistent with a growing body of literature documenting the rise of early-onset CRC in the U.S. In particular, the EAPC for ASMR among males aged 25–49 was +0.24%, indicating a persistent upward trend over the past three decades and underscoring an urgent public health challenge. It is important to emphasize that this increase should not be attributed to red meat intake alone. Early-onset CRC is widely recognized as a multifactorial phenomenon, influenced by a complex interplay of dietary, metabolic, behavioral, and biological factors. High red meat consumption may represent one contributing component within this broader risk landscape, particularly given evidence that meat intake peaks among U.S. adults aged 20–49, with the highest red meat consumption observed in middle-aged groups (11, 24). However, other established contributors—including rising obesity prevalence, physical inactivity, shifts in dietary patterns toward ultra-processed foods, alterations in the gut microbiome, and birth cohort effects—are also thought to play substantial roles in driving early-onset CRC trends. Furthermore, changes in screening practices complicate the interpretation of mortality patterns in younger adults. A 7-year retrospective cohort study in Florida demonstrated that CRC screening among individuals aged 45–49 reduced incidence by approximately 50% compared with unscreened populations, supporting the effectiveness of earlier detection (25). In recognition of these trends, the American Cancer Society lowered the recommended starting age for CRC screening from 50 to 45 years in 2018. Within the context of the GBD comparative risk assessment framework, our estimates reflect the population-level burden attributable to high red meat intake under modeled assumptions, rather than the full spectrum of interacting risk factors underlying early-onset CRC. As such, the observed increase among younger adults should be interpreted as the result of multiple converging influences, with red meat intake representing only one modifiable component.

In our study, males consistently exhibited higher CRC mortality and DALYs rate attributable to diet high in red meat compared to females across all age groups. Several factors may contribute to this sex specific difference. First, red meat consumption is generally higher among males than females in the U.S., which directly increases their attributable dietary risk. Second, differences in CRC screening uptake may also play a role–studies have shown that females are more likely to participate in preventive health services including colonoscopy than males. Third, hormonal differences may contribute to biological vulnerability; for instance, estrogen is thought to exert protective effects on the colorectal mucosa, potentially lowering CRC risk in pre-menopausal women (26). Moreover, lifestyle behaviors such as alcohol intake, physical inactivity, and smoking–often more prevalent among males–may interact synergistically with dietary risks to elevate CRC susceptibility. In addition to these sex–based mechanisms, evidence from other research highlights that meat consumption behaviors differ markedly across social strata, age groups, normative settings and context–for example, higher meat intake is associated with middle aged groups, lower educational level, lower income, stronger adherence to traditional gender or social norms that equate meat eating with masculinity, and dining contexts with higher availability of meat choices (27). These social contextual differences likely amplify the male excess in red meat related CRC burden, especially in populations with lower socio–economic status or in high consumption age bands. Such findings underscore the need for tailored public health strategies that account not only for sex, but also for age, socioeconomic status, and cultural normative influences when designing red meat reduction and CRC prevention programmes.

Although this study focused on red meat, it is important to note that processed red meat–such as bacon, sausages, and deli meats–is considered to have a stronger and more direct association with CRC risk. Several meta-analyses and IARC evaluations have classified processed meats as Group 1 carcinogens, and the magnitude of relative risk per gram consumed is often higher than for unprocessed meat. While GBD categorizes these as separate risk factors, future analyses could help quantify the joint or comparative burden attributable to each, especially in states or subgroups with particularly high intake of processed meat products.

Our decomposition analysis provides important insight into the apparent paradox observed in the U.S. CRC burden attributable to diet high in red meat. Although age-standardized mortality and DALY rates have declined over time, the absolute number of deaths has continued to increase. This divergence reflects the dominant influence of demographic momentum, whereby population growth and population aging exert upward pressure on the total burden that outweighs improvements in per-capita risk. Specifically, the negative contribution of epidemiological change suggests that, on average, the individual risk of CRC mortality attributable to high red meat intake has decreased, likely reflecting advances in screening, earlier detection, improved treatment, and broader public health efforts. However, these gains have been more than offset by the expanding size of the population and the rapid growth of older age groups, in whom CRC risk is intrinsically higher. As a result, even effective prevention and risk reduction strategies may coexist with a rising absolute disease burden at the population level. This pattern mirrors trends observed in other high-SDI regions and underscores a fundamental challenge for health systems: reductions in age-standardized rates do not necessarily translate into reductions in total health burden when demographic forces are strong. In the U.S. context, these demographic pressures may interact with persistent socioeconomic inequalities that influence CRC diagnosis and outcomes (28). For example, uneven distribution of CRC screening resources across states, particularly in socioeconomically disadvantaged regions such as Mississippi, has been shown to be associated with worse CRC outcomes (29). Taken together, these findings highlight that sustaining declines in per-capita risk is necessary but insufficient to curb the overall CRC burden. Public health strategies must therefore account for demographic momentum by coupling prevention and screening efforts with policies that expand healthcare access and address structural determinants of diet and health, particularly in aging and high-risk populations.

These findings also reveal a critical public health paradox: although the negative contribution of epidemiological change indicates that per-capita mortality attributable to high red meat intake has declined–likely due to improvements in CRC screening, diagnosis, treatment, and possibly dietary shifts–the gains have been outpaced by demographic drivers. Specifically, the combined effects of population growth and aging have resulted in an overall increase in absolute deaths and DALYs, despite improvements in individual-level risk reduction. This underscores a key challenge for health systems: effective per-capita prevention does not necessarily translate into reduced total burden unless demographic momentum is also addressed. Thus, to meaningfully alleviate the system-wide impact of CRC, strategies must not only sustain prevention gains but also adapt to the pressures of an expanding and aging population. Without such adjustments, total CRC burden may continue to rise even in the face of improved health behaviors and clinical care. While high red meat consumption is an important modifiable dietary risk factor, it represents only one component of the broader CRC risk landscape. Obesity, physical inactivity, low dietary fiber intake, and alcohol consumption also contribute significantly to CRC burden, and their interactions with red meat intake may be synergistic. For example, rising obesity rates–particularly among younger adults–may compound the inflammatory and metabolic pathways involved in carcinogenesis. Additionally, birth cohort effects and shifts in the gut microbiome due to Westernized diets may also play a role in the increasing CRC burden in younger populations. A more integrated approach to risk reduction should thus consider multiple lifestyle and dietary factors in tandem.

Importantly, this analysis demonstrates how population-level, subnational estimates of diet-attributable colorectal cancer burden can be used to inform more targeted public health decision-making. By identifying states and age groups with disproportionate burden, these findings provide a quantitative basis for prioritizing prevention efforts, guiding screening outreach, and aligning dietary risk reduction initiatives with broader cancer control strategies. While the estimates presented here are model-based and should not be interpreted causally, they nonetheless offer a valuable framework for integrating dietary risk into colorectal cancer prevention planning at both national and state levels.

Several important limitations of this study warrant careful consideration and more critical interpretation of the findings. First, within the GBD 2021 framework, “diet high in red meat” is restricted to unprocessed mammalian muscle meats and explicitly excludes processed meat products. This represents a substantive limitation rather than a minor definitional issue. Processed meats have been consistently shown to confer a stronger and more direct association with colorectal cancer risk, and are classified as Group 1 carcinogens by the International Agency for Research on Cancer. Their exclusion likely results in a systematic underestimation of the total diet-attributable colorectal cancer burden, particularly in populations and states where consumption of processed meats is high. Moreover, because processed and unprocessed meats often co-occur within dietary patterns, isolating unprocessed red meat may incompletely capture real-world exposure profiles, thereby attenuating the apparent magnitude of dietary risk. Second, the comparative risk assessment approach used in GBD does not allow modeling of dietary substitutions. In reality, reductions in red meat intake are typically accompanied by increases in other foods (e.g., poultry, fish, legumes, whole grains, or refined carbohydrates), each of which may have distinct and sometimes opposing effects on colorectal cancer risk. The inability to account for these substitutions limits causal interpretability and may oversimplify the health implications of dietary change. Consequently, the estimated burden attributable to high red meat intake should be interpreted as a counterfactual comparison against a theoretical minimum exposure level, rather than as an estimate of the health impact of realistic dietary transitions. Third, this analysis evaluates red meat intake as a single, isolated risk factor and does not account for interactions with other dietary, metabolic, or behavioral risks. Colorectal carcinogenesis is a multifactorial process influenced by obesity, physical inactivity, alcohol consumption, low fiber intake, metabolic dysfunction, and gut microbiome alterations. These factors may interact synergistically with red meat intake, particularly in younger cohorts and socioeconomically disadvantaged populations. The absence of multi-risk interaction modeling may therefore obscure important effect modification and partially explain observed age- and state-level heterogeneity in attributable burden. In addition, the ecological and model-based nature of GBD estimates precludes individual-level inference and causal interpretation. The attributable fractions represent hypothetical reductions in population burden under modeled exposure shifts, rather than observed epidemiological effects. Furthermore, uncertainty in dietary exposure estimation, reliance on heterogeneous data sources, and assumptions regarding theoretical minimum risk exposure levels introduce additional uncertainty. Collectively, these limitations suggest that our findings should be interpreted as population-level estimates within a defined modeling framework, and underscore the need for future studies integrating individual-level data, dietary pattern analysis, substitution modeling, and multi-risk interaction frameworks.

## 5 Conclusion

In conclusion, although age-standardized rates of colorectal cancer attributable to high red meat intake have declined in the U.S. over the past three decades, the increasing burden among younger adults and the persistence of marked state-level disparities remain major public health concerns. These findings suggest that colorectal cancer prevention efforts may benefit from greater geographic and demographic targeting, with particular attention to high-burden states and younger populations experiencing rising risk. While this study does not evaluate specific interventions, it provides population-level evidence that may support the alignment of dietary risk reduction strategies, colorectal cancer screening policies, and state-level public health planning. Future research incorporating individual-level data, dietary substitution modeling, and multi-risk frameworks will be critical for translating these population estimates into more precise and effective prevention strategies.

## Data Availability

The original contributions presented in the study are included in the article/Supplementary material, further inquiries can be directed to the corresponding author.
