# Supplementary material for: Burden of colon and rectum cancer attributable to a diet high in red meat in the United States, 1990–2021
Source: Front Nutr. 2026 Mar 24;13:1683427. doi: 10.3389/fnut.2026.1683427 (PMC13053255; doi:10.3389/fnut.2026.1683427)
Supplement: Supplementary file 1 [file Table_1.docx]

Addtional table 1 Death number, ASMR, and ASDR of colon and rectum cancer attribute to diet high in red meat in 51 states of the United States in 2021.

| States | Deaths number (95% UI) | ASMR (95% UI) | ASDR (95% UI) |
| --- | --- | --- | --- |
| Alabama | 223.5 (-0.1 to 450.8) | 2.55 (0 to 5.1) | 65.8 (0 to 133.5) |
| Alaska | 21.2 (0 to 43) | 2.08 (0 to 4.3) | 50.6 (0 to 103) |
| Arizona | 251.6 (-0.1 to 516.8) | 1.96 (0 to 4) | 49.6 (0 to 101.8) |
| Arkansas | 133.3 (-0.1 to 270.7) | 2.54 (0 to 5.2) | 64.9 (0 to 132.1) |
| California | 1172.2 (-0.7 to 2353.3) | 1.78 (0 to 3.6) | 44.8 (0 to 90.1) |
| Colorado | 167.4 (-0.1 to 341.2) | 1.76 (0 to 3.6) | 42 (0 to 86.9) |
| Connecticut | 124 (-0.1 to 247.5) | 1.7 (0 to 3.4) | 40.7 (0 to 80.9) |
| Delaware | 40.5 (0 to 83.1) | 2.1 (0 to 4.3) | 51.6 (0 to 106.8) |
| District of Columbia | 21.8 (0 to 43.9) | 2.21 (0 to 4.4) | 55.6 (0 to 110.7) |
| Florida | 868.3 (-0.7 to 1772.3) | 1.97 (0 to 4) | 51.4 (0 to 104.8) |
| Georgia | 382.5 (-0.2 to 776.1) | 2.27 (0 to 4.6) | 57.2 (0 to 115.9) |
| Hawaii | 54.2 (0 to 108.1) | 1.85 (0 to 3.7) | 46.9 (0 to 93.5) |
| Idaho | 57.3 (0 to 114) | 1.86 (0 to 3.7) | 44 (0 to 86) |
| Illinois | 494.7 (-0.3 to 1018.3) | 2.19 (0 to 4.5) | 53.5 (0 to 109.6) |
| Indiana | 275.8 (-0.1 to 554.7) | 2.37 (0 to 4.8) | 58.1 (0 to 116.5) |
| Iowa | 132.4 (-0.1 to 259.7) | 2.15 (0 to 4.2) | 51 (0 to 99.6) |
| Kansas | 109.3 (-0.1 to 226.2) | 2.15 (0 to 4.4) | 52.8 (0 to 107.3) |
| Kentucky | 211.1 (-0.1 to 436.7) | 2.69 (0 to 5.5) | 67.8 (0 to 140.5) |
| Louisiana | 210.3 (-0.1 to 421) | 2.79 (0 to 5.5) | 71 (-0.1 to 139.2) |
| Maine | 62.2 (0 to 127.1) | 2.06 (0 to 4.2) | 49.4 (0 to 100.4) |
| Maryland | 217.4 (-0.1 to 421.6) | 2.02 (0 to 3.9) | 51 (0 to 99) |
| Massachusetts | 227.7 (-0.1 to 462.6) | 1.71 (0 to 3.5) | 40.4 (0 to 82.7) |
| Michigan | 380.7 (-0.2 to 784) | 1.99 (0 to 4.1) | 49.2 (0 to 101.4) |
| Minnesota | 192.2 (-0.1 to 389.6) | 1.84 (0 to 3.7) | 42.9 (0 to 87) |
| Mississippi | 145.2 (-0.1 to 295.6) | 2.96 (0 to 6) | 77.2 (-0.1 to 156.3) |
| Missouri | 259.3 (-0.1 to 523.8) | 2.31 (0 to 4.6) | 57.7 (0 to 117.9) |
| Montana | 43.1 (0 to 89.3) | 1.96 (0 to 4.1) | 47 (0 to 95.7) |
| Nebraska | 79.7 (0 to 158.3) | 2.28 (0 to 4.5) | 54.2 (0 to 107.5) |
| Nevada | 109.8 (0 to 218.2) | 2.14 (0 to 4.3) | 52.2 (0 to 103.9) |
| New Hampshire | 51.7 (0 to 104.4) | 1.84 (0 to 3.7) | 42.6 (0 to 85.2) |
| New Jersey | 323.2 (-0.1 to 650.8) | 1.88 (0 to 3.8) | 45.7 (0 to 91) |
| New Mexico | 76.1 (0 to 157.3) | 1.98 (0 to 4.1) | 52.2 (0 to 107.3) |
| New York | 649.5 (-0.3 to 1318.5) | 1.75 (0 to 3.5) | 43.7 (0 to 86.2) |
| North Carolina | 389.6 (-0.2 to 798.8) | 2.11 (0 to 4.3) | 53 (0 to 107.6) |
| North Dakota | 27.5 (0 to 55.2) | 1.92 (0 to 3.8) | 46.4 (0 to 92) |
| Ohio | 509.1 (-0.2 to 1004.6) | 2.31 (0 to 4.6) | 56.4 (0 to 111.5) |
| Oklahoma | 160.5 (-0.1 to 312.1) | 2.43 (0 to 4.7) | 61.5 (0 to 120) |
| Oregon | 154.4 (-0.1 to 314.5) | 1.86 (0 to 3.8) | 44.7 (0 to 91.5) |
| Pennsylvania | 548.2 (-0.3 to 1142) | 2.12 (0 to 4.4) | 51.5 (0 to 106.3) |
| Rhode Island | 39.8 (0 to 81.1) | 1.83 (0 to 3.8) | 42.4 (0 to 86.8) |
| South Carolina | 210.4 (-0.1 to 426.4) | 2.29 (0 to 4.6) | 58.7 (0 to 118.1) |
| South Dakota | 33.2 (0 to 67) | 1.99 (0 to 4) | 48.6 (0 to 96.6) |
| Tennessee | 297.8 (-0.2 to 587.3) | 2.47 (0 to 4.9) | 62.6 (0 to 124) |
| Texas | 942.3 (-0.6 to 1905.6) | 2.22 (0 to 4.5) | 56.5 (0 to 114.3) |
| Utah | 71.1 (0 to 142.2) | 1.73 (0 to 3.5) | 40 (0 to 79.3) |
| Vermont | 26.1 (0 to 53.1) | 1.9 (0 to 3.8) | 45.1 (0 to 92.1) |
| Virginia | 308.2 (-0.1 to 621) | 2.08 (0 to 4.2) | 51.4 (0 to 102.4) |
| Washington | 238.3 (-0.1 to 479.8) | 1.75 (0 to 3.5) | 42 (0 to 85) |
| West Virginia | 98.1 (0 to 193.2) | 2.72 (0 to 5.4) | 70.4 (0 to 138.3) |
| Wisconsin | 208.7 (-0.1 to 418.8) | 1.87 (0 to 3.7) | 44.2 (0 to 88.4) |
| Wyoming | 20.5 (0 to 42.4) | 2 (0 to 4.1) | 48.3 (0 to 99.3) |

Note: ASMR, age-standardized mortality rate; ASDR, age-standardized DALYs rate; DALYs, disability-adjusted life years; UI, uncertainty interval.
