# Supplementary material for: Burden of colon and rectum cancer attributable to a diet high in red meat in the United States, 1990–2021
Source: Front Nutr. 2026 Mar 24;13:1683427. doi: 10.3389/fnut.2026.1683427 (PMC13053255; doi:10.3389/fnut.2026.1683427)
Supplement: Supplementary file 2 [file Table_2.docx]

| Measure  Additional table 2 Decomposition analysis of deaths and DALYs of colon and rectum cancer attribute to diet high in red meat in global, regions with 5 SDI quintiles, and United States. | Location | Sex | Aging (%%) | Population (%%) | Epidemiological change (%%) |
| --- | --- | --- | --- | --- | --- |
| DALYs | Global | Both | 516565.6 (35.44%) | 1643693.9 (112.77%) | -702753.27 (-48.22%) |
|  |  | Female | 214624.1 (44.25%) | 735860 (151.72%) | -465479.18 (-95.97%) |
|  |  | Male | 314723 (32.36%) | 906179.9 (93.18%) | -248401.6 (-25.54%) |
|  | High SDI | Both | 238190 (113.97%) | 342583.9 (163.92%) | -371780.18 (-177.89%) |
|  |  | Female | 96628 (198.22%) | 144636.9 (296.71%) | -192517.27 (-394.93%) |
|  |  | Male | 155680.4 (97.15%) | 201177.4 (125.54%) | -196611.74 (-122.69%) |
|  | High-middle SDI | Both | 196354.3 (45.4%) | 387573.2 (89.61%) | -151400.27 (-35%) |
|  |  | Female | 78013.1 (60.61%) | 165585.7 (128.65%) | -114888.27 (-89.26%) |
|  |  | Male | 127791.3 (42.06%) | 223239.8 (73.48%) | -47214.39 (-15.54%) |
|  | Middle SDI | Both | 165850.1 (27.82%) | 449137.7 (75.34%) | -18836.66 (-3.16%) |
|  |  | Female | 70787.2 (34.9%) | 195470.1 (96.38%) | -63445.16 (-31.28%) |
|  |  | Male | 95615.3 (24.31%) | 251517 (63.94%) | 46206.76 (11.75%) |
|  | Low-middle SDI | Both | 15361.6 (9.04%) | 118189 (69.54%) | 36404.63 (21.42%) |
|  |  | Female | 9126.7 (11.25%) | 60934.4 (75.13%) | 11045.23 (13.62%) |
|  |  | Male | 6087.7 (6.85%) | 57321.2 (64.52%) | 25440.04 (28.63%) |
|  | Low SDI | Both | -3035.4 (-6.28%) | 60620.9 (125.36%) | -9227.85 (-19.08%) |
|  |  | Female | -678.7 (-2.94%) | 28406.9 (122.98%) | -4629.46 (-20.04%) |
|  |  | Male | -2524.1 (-9.99%) | 32123.1 (127.17%) | -4340.02 (-17.18%) |
|  | United States of America | Both | 58793.8 (154.33%) | 93843.1 (246.33%) | -114540.1 (-300.66%) |
|  |  | Female | 21909.8 (251.78%) | 41021 (471.4%) | -54228.91 (-623.19%) |
|  |  | Male | 39546.1 (134.53%) | 53534.5 (182.12%) | -63685.66 (-216.66%) |
| Deaths | Global | Both | 29955 (43.59%) | 68535.5 (99.73%) | -29767.8 (-43.32%) |
|  |  | Female | 13190.2 (51.79%) | 32409.3 (127.25%) | -20130.81 (-79.04%) |
|  |  | Male | 17566.4 (40.61%) | 36084.4 (83.42%) | -10396.7 (-24.04%) |
|  | High SDI | Both | 15593.5 (111.21%) | 16233 (115.77%) | -17804.58 (-126.98%) |
|  |  | Female | 6997.1 (145.26%) | 7372 (153.04%) | -9552.15 (-198.3%) |
|  |  | Male | 9509.1 (103.3%) | 8950.7 (97.24%) | -9254.77 (-100.54%) |
|  | High-middle SDI | Both | 10968.1 (50.65%) | 15966.7 (73.74%) | -5281.26 (-24.39%) |
|  |  | Female | 4627.9 (60.68%) | 7202.3 (94.44%) | -4203.9 (-55.12%) |
|  |  | Male | 6901.5 (49.2%) | 8802.3 (62.75%) | -1676.63 (-11.95%) |
|  | Middle SDI | Both | 8359.8 (33.48%) | 16433.7 (65.81%) | 179.32 (0.72%) |
|  |  | Female | 3655.7 (40.01%) | 7394.1 (80.92%) | -1912.28 (-20.93%) |
|  |  | Male | 4744.4 (29.96%) | 8975.4 (56.68%) | 2115.55 (13.36%) |
|  | Low-middle SDI | Both | 795.2 (12.71%) | 4147.6 (66.32%) | 1311.45 (20.97%) |
|  |  | Female | 478.8 (15.78%) | 2142.7 (70.62%) | 412.48 (13.6%) |
|  |  | Male | 309.6 (9.61%) | 2007.7 (62.35%) | 902.93 (28.04%) |
|  | Low SDI | Both | -99 (-5.69%) | 2103.7 (120.88%) | -264.42 (-15.19%) |
|  |  | Female | -15.9 (-1.94%) | 969.5 (117.85%) | -130.9 (-15.91%) |
|  |  | Male | -90.4 (-9.85%) | 1130.5 (123.2%) | -122.51 (-13.35%) |
|  | United States of America | Both | 2947.5 (225.84%) | 4211.4 (322.68%) | -5853.76 (-448.51%) |
|  |  | Female | 1112.3 (425.34%) | 1962.1 (750.33%) | -2812.89 (-1075.67%) |
|  |  | Male | 2011.4 (192.73%) | 2273.2 (217.82%) | -3240.96 (-310.54%) |

Note: DALYs, disability-adjusted life years; SDI, socio-demographic index.
